# Supplementary material for: Immunomodulation of Natural Killer Cell Function by Ribavirin Involves TYK-2 Activation and Subsequent Increased IFN-γ Secretion in the Context of In Vitro Hepatitis E Virus Infection
Source: Cells. 2023 Jan 31;12(3):453. doi: 10.3390/cells12030453 (PMC9913562; doi:10.3390/cells12030453)
Supplement: Supplementary file 1 [file cells-12-00453-s001.zip › cells-2142545-supplementary.pdf]

Supplementary Figure S1

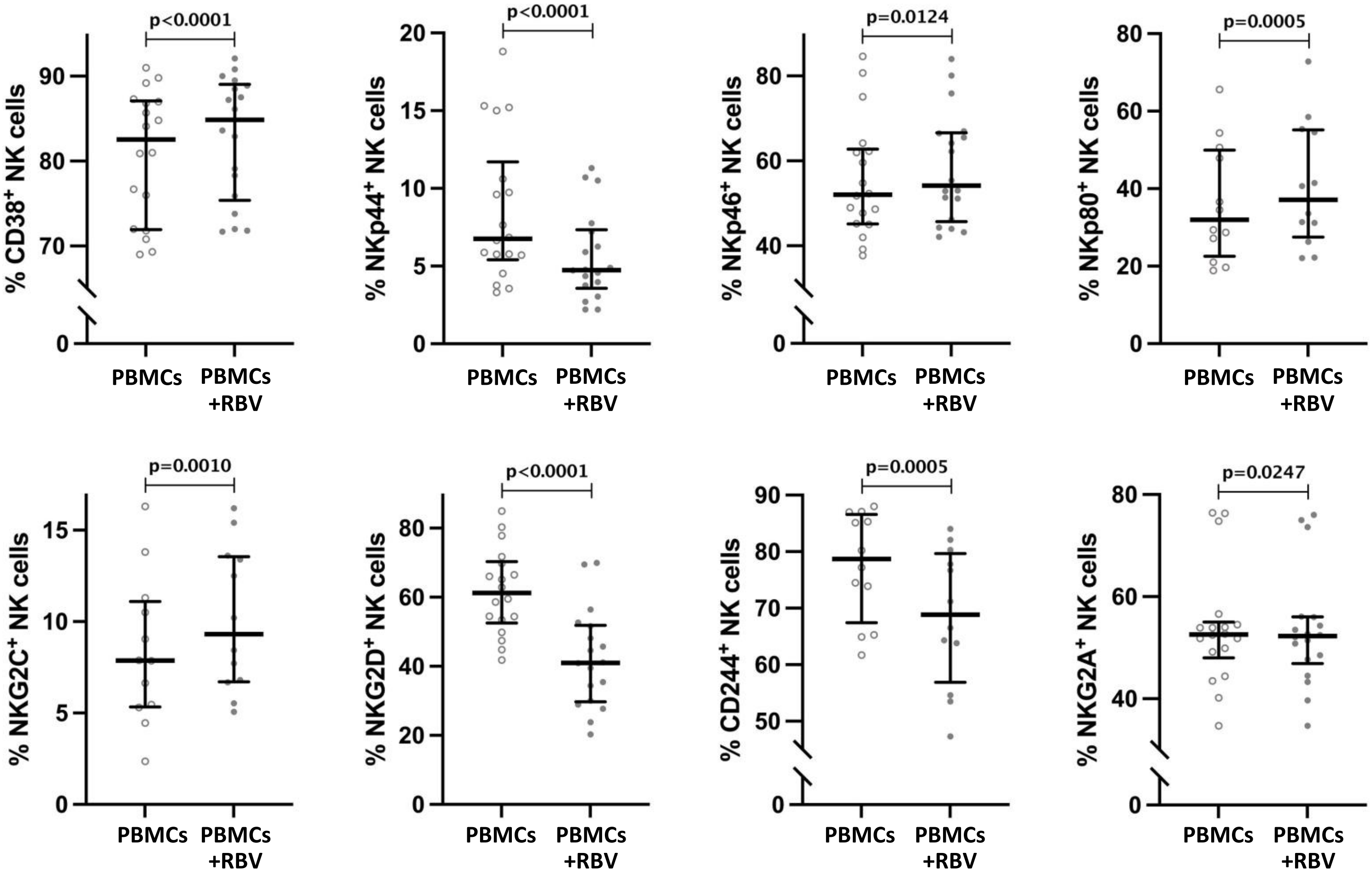

Supplementary Figure S2

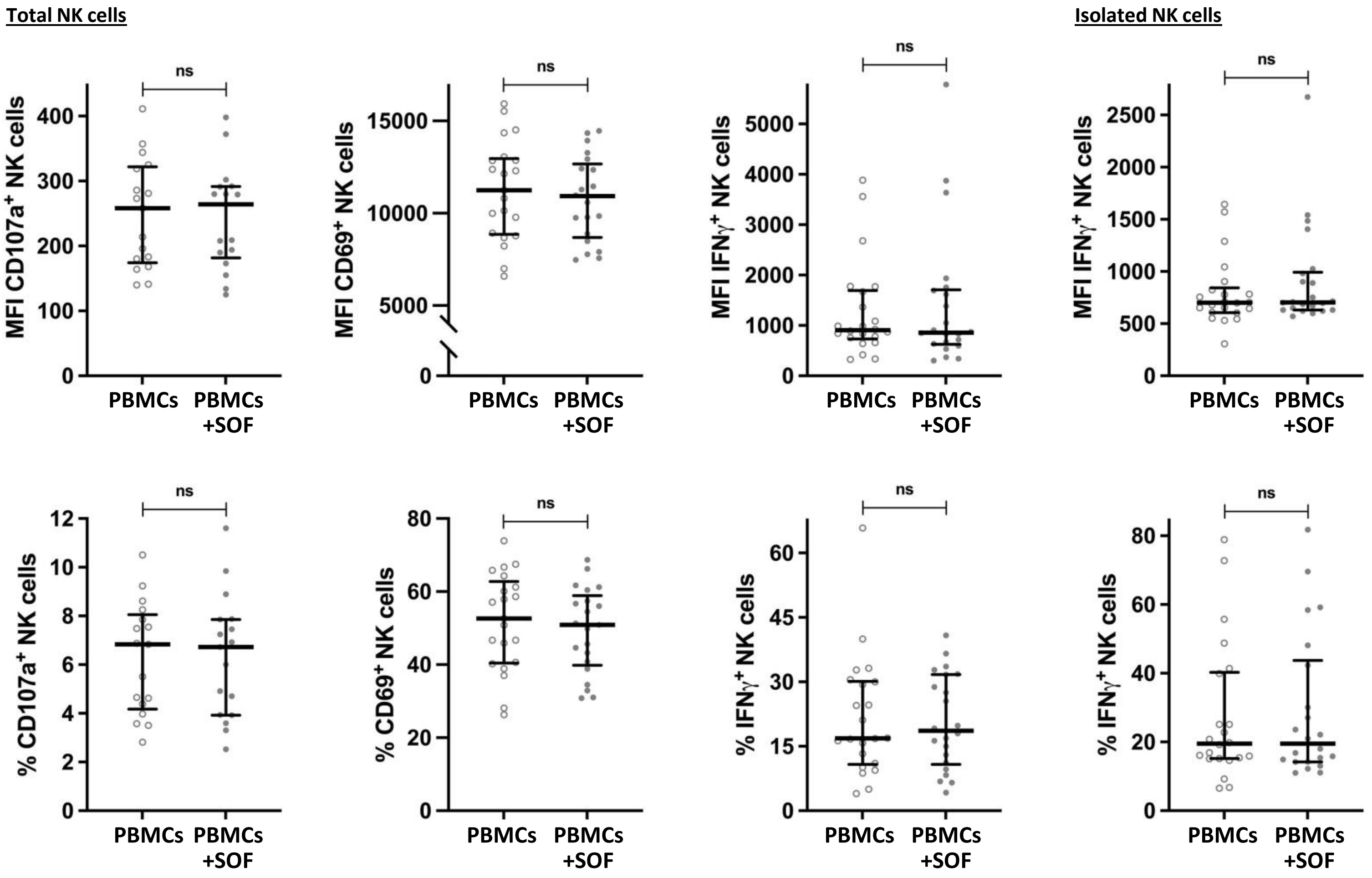

Supplementary Figure S3

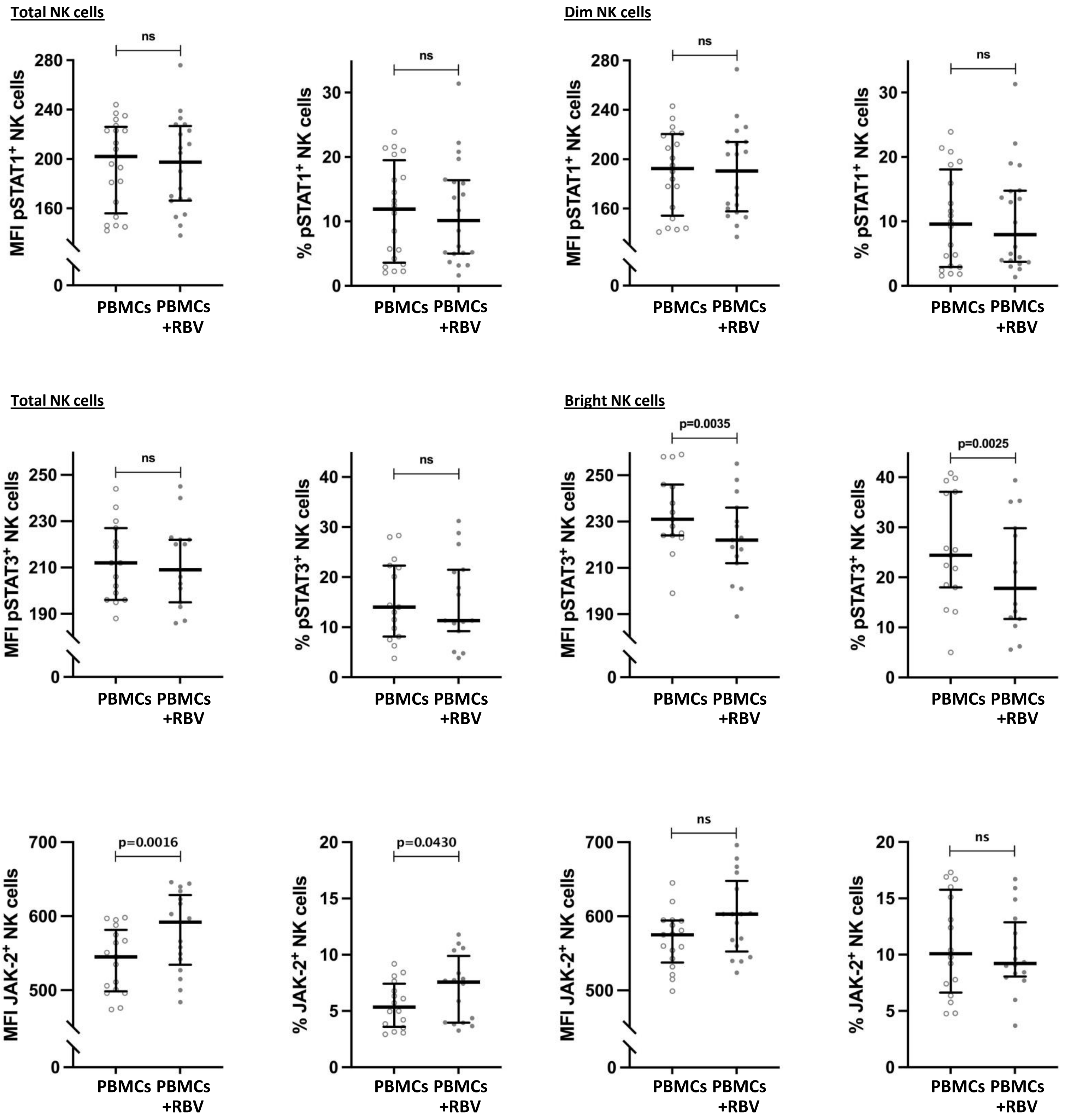

Supplementary Figure S4

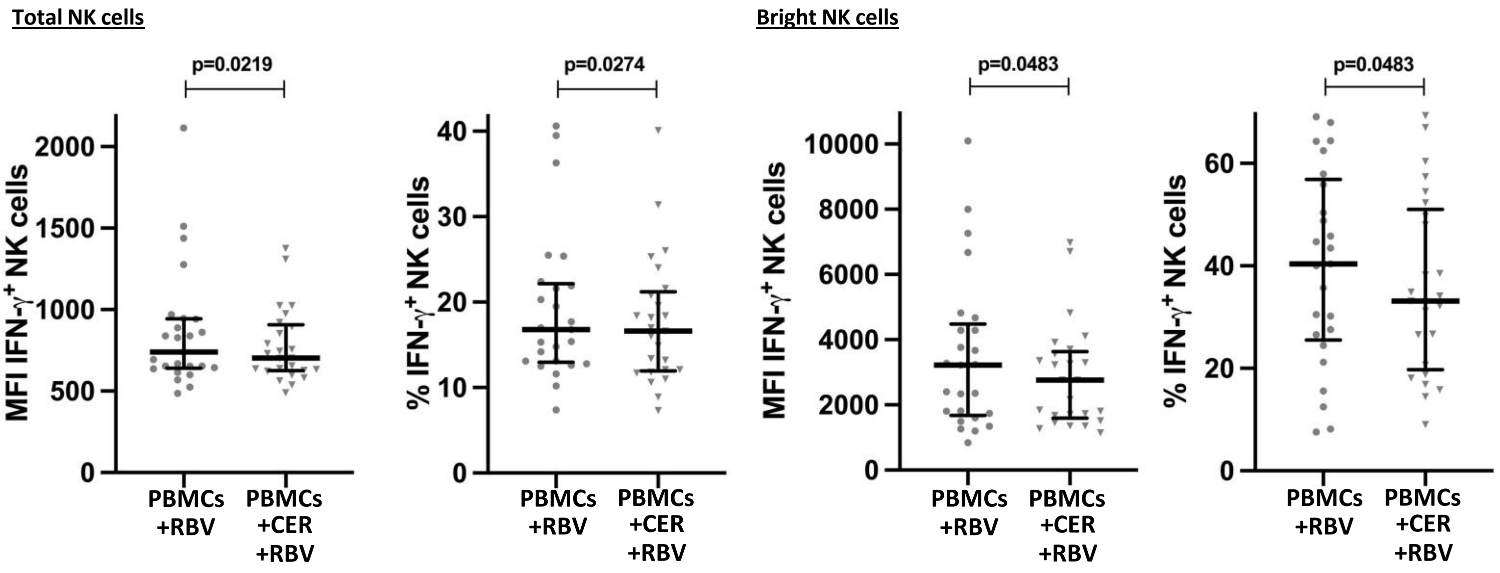

Supplementary Figure S1:

Flow cytometric analysis of NK cells and involved receptors under RBV treatment. Expression of affected NK cell receptors shown by frequencies. (n=18 and n=12) Appearance: median with interquartile range, statistical analysis: Wilcoxon matched-pairs signed rank test.

Supplementary Figure S2:

Flow cytometric analysis of NK cells under SOF treatment. MFI and frequencies compared. NK cell degranulation analyzed by CD107a expression upon K562 stimulation. (n=17) Activation of NK cells represented by CD69 expression upon IL-12/IL-15 stimulation. (n=21) Analysis of IFN-γ upon IL-12/IL-15 stimulation as a major indicator for NK cell cytokine production (n=22) compared to isolated NK cells. (n=22) Appearance: median with interquartile range, statistical analysis: Wilcoxon matched-pairs signed rank test.

Supplementary Figure S3:

Flow cytometric analysis of IL-12 signaling in NK cells under RBV treatment. MFI and frequencies compared. Expression of pSTAT1 (n=20), pSTAT3 (n=15) and JAK-2 (n=17) upon IL-12/IL-15 stimulation. Appearance: median with interquartile range, statistical analysis: Wilcoxon matched-pairs signed rank test.

Supplementary Figure S4:

Flow cytometric analysis of NK cells under RBV treatment, selectively TYK-2 inhibited by cerdulatinib. MFI and frequencies compared. Analysis of IFN-γ upon IL-12/IL-15 stimulation. (n=25) Appearance: median with interquartile range, statistical analysis: Wilcoxon matched-pairs signed rank test.
